# Reliability of dynamic susceptibility contrast perfusion metrics in pre- and post-treatment glioma

**DOI:** 10.1186/s40644-022-00466-2

**Published:** 2022-06-17

**Authors:** Valentina Kouwenberg, Lusien van Santwijk, Frederick J. A. Meijer, Dylan Henssen

**Affiliations:** Department of Medical Imaging, Radboud University Medical Center, Geert Grooteplein Zuid 10, 6525 EZ Nijmegen, The Netherlands

**Keywords:** Dynamic susceptibility contrast magnetic resonance (DSC-MR) perfusion imaging, Glioma, Interobserver variability, Region of interest

## Abstract

**Background:**

In neuro-oncology, dynamic susceptibility contrast magnetic resonance (DSC-MR) perfusion imaging emerged as a tool to aid in the diagnostic work-up and to surveil effectiveness of treatment. However, it is believed that a significant variability exists with regard to the measured in DSC-MR perfusion parameters. The aim of this study was to assess the observer variability in measured DSC-MR perfusion parameters in patients before and after treatment. In addition, we investigated whether region-of-interest (ROI) shape impacted the observer variability.

**Materials and methods:**

Twenty non-treated patients and a matched group of twenty patients post-treatment (neurosurgical resection and post-chemoradiotherapy) were included. Six ROIs were independently placed by three readers: circular ROIs and polygonal ROIs covering 1) the tumor hotspot; 2) the peritumoral region (T2/FLAIR-hyperintense region) and 3) the whole tumor region. A two-way random Intra-class coefficient (ICC) model was used to assess variability in measured DSC-MRI perfusion parameters. The perfusion metrics as assessed by the circular and the polygonal ROI were compared by use of the dependent T-test.

**Results:**

In the non-treated group, circular ROIs showed good–excellent overlap (ICC-values ranging from 0.741–0.963) with the exception of those representing the tumor hotspot. Polygonal ROIs showed lower ICC-values, ranging from 0.113 till 0.856. ROI-placement in the posttreatment group showed to be highly variable with a significant deterioration of ICC-values. Furthermore, perfusion metric assessment in similar tumor regions was not impacted by ROI shape.

**Discussion:**

This study shows that posttreatment quantitative interpretation of DSC-MR perfusion imaging is highly variable and should be carried out with precaution. Pretreatment assessment of DSC-MR images, however, could be carried out be a single reader in order to provide valid data for further analyses.

## Highlights

• DSC-MR perfusion imaging measurements in non-treated glioma is highly reliable between readers, even readers with little experience.

• DSC-MR perfusion imaging measurements in treated glioma is show to be inconsistent between readers.

• When using DSC-MR perfusion imaging as a quantitative surveillance tool for the recurrence of glioma after treatment, double-reading should be preferred.

## Introduction

In neuro-oncology, dynamic susceptibility contrast magnetic resonance imaging (DSC-MRI) can play an important role in non-invasive tumor grading, prediction of treatment response [1]. In the diagnostic work-up, DSC-MRI can be used for characterisation of glioma genotype, as it is known that genetic differences in glioma subtypes correlate with the glioma vasculature. This results in specific perfusion profiles of different subtypes of glioma, as described in a recent review and meta-analysis of our group [2]. However, DSC-MRI has a major role to play in the post-treatment setting, especially with regard to the differentiation between true early tumor progression and pseudoprogression. Due to the infiltrative nature of gliomas, total resection is almost impossible and residual glioma cells can further infiltrate the brain tissue surrounding the resection cavity. This results in an enhancing lesion on pre- and post-contrast T1-weighted images. Although these characteristics are generally reliable to assess residual or recurring neoplasm, an important imaging dilemma exist. Different treatment methods (i.e., chemotherapy and radiation) increase the permeability of the vascular walls which leads to contrast-leakage. This leakage can also be observed as a new enhancing lesion, though is called pseudoprogression. Therefore, differentiation between early tumor progression and pseudoprogression is poor with conventional MR images alone [3]. It has been reported that implementation of either DSC-MRI in routine radiological follow-up of glioblastoma can aid the detection of tumour recurrence [4], although varying practices complicate further clinical implementation [5–7].

**Fig. 1:**
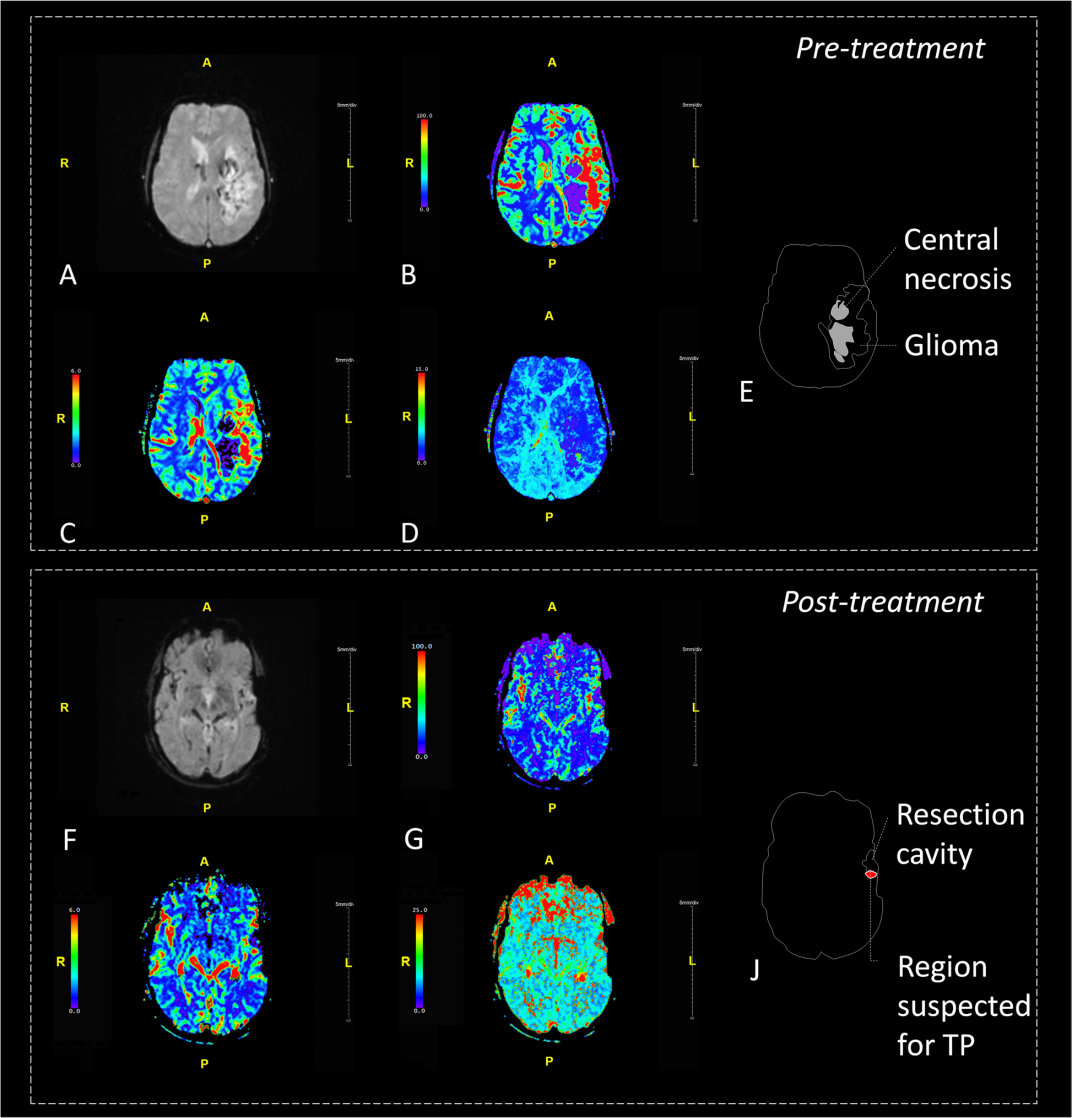
Exemplary images of DSC-MR perfusion images. First panel shows a multicompartimental glioma (WHO grade IV) located in the left hemisphere with known irregular enhancement located in the left temporal occipital and frontal lobes. The lesion contains areas of central necrosis and shows focally increased perfusion. Second panel shows status after resection of a glioma (WHO grade IV) located in the left frontal lobe. At this level, no residual tumor is observed on the derived perfusion images. **A** DSC-MR perfusion imaging source data; **B** CBF map; **C** CBV map; **D** TTP map; **E** Schematic representation of image; **F** DSC-MR perfusion imaging source data; **G** CBF map; **H** CBV map; **I** TTP map; **J** Schematic representation of regions. DSC-MRI = dynamic susceptibility contrast magnetic resonance imaging; CBV = cerebral blood volume; ICC = intraclass correlation coefficient; CBF = cerebral blood flow; TTP = time-to-peak; WHO = World Health Organization

The technique of DSC-MRI relies on the susceptibility induced signal loss on T2*-weighted sequences, resulting from the passage of a bolus of gadolinium-based contrast agent. The most commonly used DSC parameter is Cerebral Blood Volume (CBV) which can be estimated [8, 9] and computed [10] based on the negative enhancement integral. Other parameters include Cerebral Blood Flow (CBF), Mean Transit Time (MTT) and Time To Peak (TTP) (Fig. 1). The estimated value of the area under the attenuation curve is proportional to the CBV but does not yield an absolute measurement. Therefore, the measurement is expressed relative to a standard reference, usually the contralateral white matter (relative CBV ratio: rCBV ratio) [11]. Overall, the rCBV ratio is an indicator of hypervascular regions and serves as the most robust parameter in DSC MRI [12].

DSC-MRI data is most commonly analyzed using region-of-interest (ROI) measurements, which remains an operator-dependent method [13–15]. ROI measurement of tumor cerebral blood volume (CBV) has shown good reproducibility [16], but nevertheless remains operator-dependent and fairly subjective, with an unavoidable component of interobserver and intra-observer variability [17, 18]. For example, repeatability and reproducibility were found to be below 50% and 10% respectively in one multicentre study [16]. However, it has been shown that a well-established image-review process can help to assess perfusion metrics more objectively. It has also been recommended to assess DSC-MRI data in the context of clinical trials by two experienced readers. In case of disagreement, an adjudicator could be consulted [16]. Albeit, this is clearly time consuming and thus impractical for clinical practice. However, much remains unknown with regard to measuring DSC-MRI data, including whether the shape of the ROI correlates with the variability in perfusion metrics. Also, as the aforementioned multicenter trial was performed on post-operative gliomas, the repeatability and reproducibility of DSC-MRI data in non-operated gliomas remains unknown. Finally, it remains unknown whether less experienced readers can provide robust results by using a well-established image-review process upfront to the assessment. Next to these unclarities, no broad consensus has been reached on the acquisition technique, post-processing algorithms, analysis methods or interpretation guidelines. Thereby, the use of DSC-MRI as an imaging biomarker is severely hampered, despite its extensive use in clinical practice [19].

This study aimed to investigate whether ROI shape impacts the repeatability and reproducibility of measured DSC-MRI metrics. The study was carried out after defining a well-established image-review protocol by three non-experienced readers. Finally, we assessed the repeatability and reproducibility of the DSC-MRI metrics in glioma patients in the pre- and post-surgical setting.

## Materials and methods

### Study group

Two groups of patients diagnosed with cerebral glioma were retrospectively included between December 2013 and August 2020. The two groups were age-matched, sex-matched and matched for histopathological diagnosis. The first group of patients underwent DSC-MR perfusion imaging prior to neurosurgical intervention, radiotherapy and/or receiving chemotherapy or anti-angiogenic therapies. Patients were included from the eTumour database, providing a case mix of tumors of different grades. The second group consisted of patients who underwent DSC-MR perfusion after neurosurgical treatment. The patients in the second group were at random included in a retrospective fashion. The (history of) use of anti-angiogenic therapies was an exclusion criterion for this group as well. Inclusion criteria for all patients comprised that patients needed to be at least 18 years old at time of imaging and that histopathological assessment needed to confirm the diagnosis of glioma WHO grade II, III or IV. Exclusion criteria were 1) extra-axial localization of the tumor (e.g., meningioma); 2) the presence of multicentric glioma; and/or 3) patients’ objection for data sharing.

### Ethical statement

The present study included data from the eTumour project. The eTumour project was approved by the ethical review boards of the participating centers and written informed consent was obtained from all included patients. Ethical review boards (ERBs) of the partners included the ERBs of Universitat Autònoma de Barcelona; Hospital Sant Joan de Déu; University of Birmingham; Fundación Lucha contra las Enfermedades Neurológicas de la Infancia; Cancer Research UK Cambridge Research Institute; Deutsche Krebsforschungzentrum Heidelberg; Medical University of Lodz; St Georges’ Hospital and Medical School; University Medical Centre Nijmegen, Radboud University; University of Nottingham; and Universidad de Valencia. Further details of the eTumour project can be found elsewhere [20].

In addition, the present study also included new post-operative data of different patients. Ethical approval for this part of the study was waived by our institutional review board (CMO region Arnhem-Nijmegen) because of the retrospective study design. All procedures performed were in accordance with the ethical standards of the institutional and/or national research committee and with the 1964 Helsinki Declaration and its later amendments or comparable ethical standards.

### MR imaging protocol

Imaging was performed on a 3 T clinical MR imaging system (*MAGNETOM Trio; Siemens, Erlangen, Germany*). The scanning protocol included an axial T2-weighted (T2w) sequence (repetition time (TR)/echo time (TR): 4.0/102 ms), an axial transverse fluid-attenuated inversion recovery (FLAIR) sequence (TR/TE 13,050/103 ms) and an axial T1-weighted (T1w) spin-echo sequence (TR/TE 2.3/4.7 ms) performed before and after intravenous administration of the gadolinium-based compound (*Dotarem; Guerbet, Paris, France*). The axial contrast enhanced T1w spin-echo sequence was performed after DSC-MRI acquisition. DSC-MRI was performed with a gradient-echo echoplanar imaging sequence. Prior to the dynamic study, a saturation prebolus of approximately 25% of the total gadolinium dose was administered as a preload. The preload served to reduce contaminating T1 effects from contrast agent leakage. DSC-MR images were acquired during the first pass of a (0.1 mmol/kg) bolus of contrast at a rate of 2.5 ml/s. Imaging parameters were: TR/TE 1,670/45 ms; FOV 230 × 230 mm; matrix 128 × 128; voxel size 1.8 × 1.8 × 5.0 mm3; intersection gap 30%; flip angle 90°; signal bandwidth 1,346 Hz/x. A total of 15 axial perfusion slides were reconstructed for each scan. DSC-MR perfusion imaging datasets were processed using Time Dependent Analysis software [21]. Motion correction was automatically performed on all images. The arterial input function was manually placed at the origin of middle cerebral artery ipsilateral to tumor location.

### DSC MRI image-review protocol

Perfusion characteristics of the tumor were assessed by manually placing multiple ROIs by three researchers independently. Two researchers (VK and LS) had little to no experience with regard to reading diagnostic radiology examinations, whereas the third researcher (DH) was a radiology resident with 3 years of experience and over 7 years of experience with regard to neuroimaging in the research setting. The researchers were trained by use of non-included test cases. Tumor ROIs on the DSC-MRI scans consisted of both circular and polygonal ROIs. As different regions can be appreciated on MR images of glioma, three subregions were distinguished. These regions comprised 1) the tumor hotspot representing the most-enhancing tumor region; 2) the peritumoral region and 3) the entire tumor(-region) (Fig. 1). The distinguishment of these three regions is less investigated in DSC-MRI investigations, though is commonly done in research focusing on the use of artificial intelligence in non-invasive prediction of the molecular status of the glioma [22, 23]. The rationale for the use of these different regions can be found in the fact that these regions are believed to have distinctly different biological properties. Also, these tumor subregions meet specific radiological criteria, facilitating their identification.

**Fig. 2:**
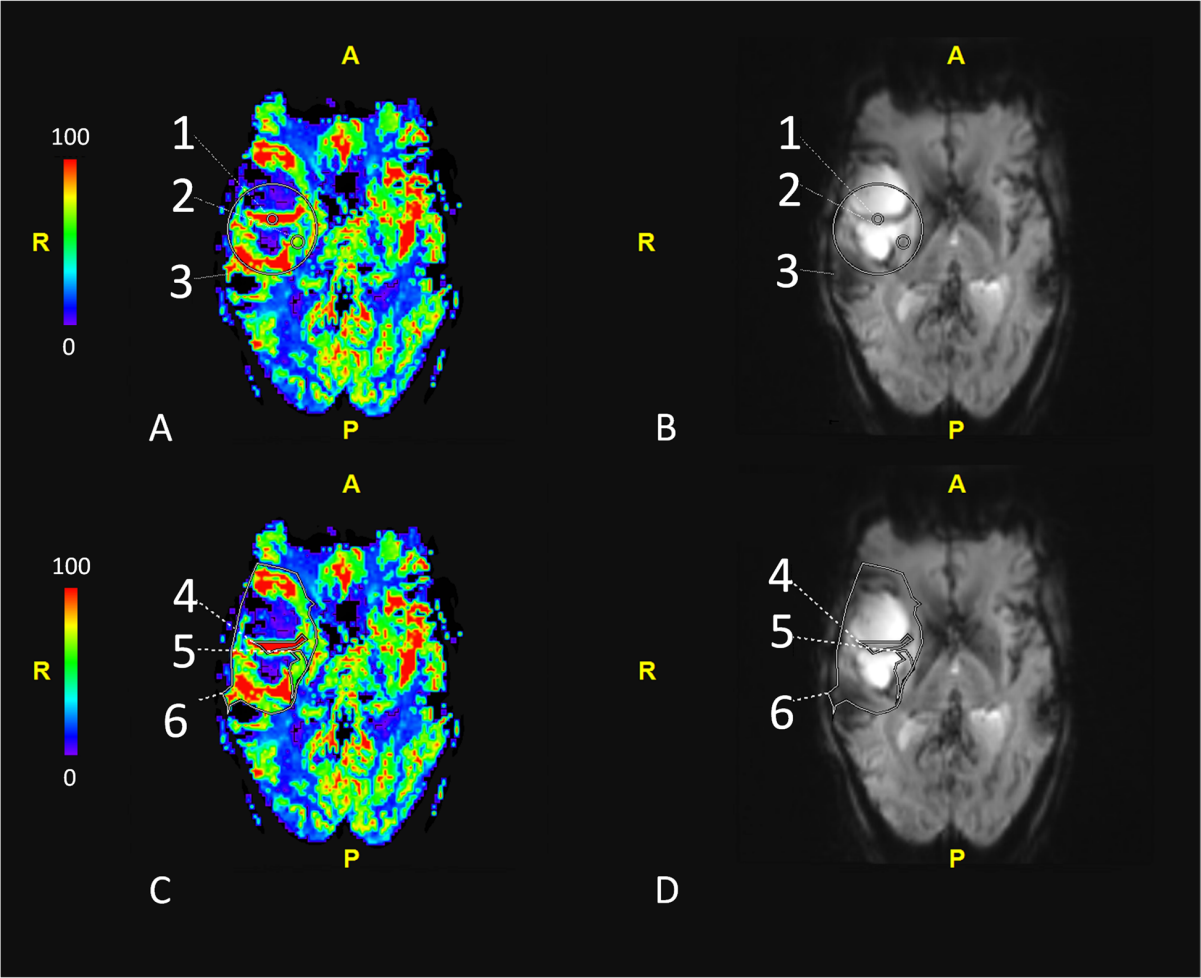
Exemplary images of the different ROIs used for this study. On the left-handed side, the CBV maps are presented (**A** and **C**); on the right-handed side, the DSC-MRI source data are presented (**B** and **D**). ROI1 = Circular ROI covering the tumor hotspot; ROI2 = Circular ROI covering the peritumoral region; ROI3 = Circular ROI covering the whole tumor region; ROI4 = Polygonal ROI covering the tumor hotspot; ROI5 = Polygonal ROI covering the peritumoral region; ROI6 = Polygonal ROI covering the whole tumor region. DSC-MRI = dynamic susceptibility contrast magnetic resonance imaging; CBV = cerebral blood volume; ROI = Region of interest

ROIs covering the whole tumor and tumor hotspot were drawn on the slide representing the largest area of increased perfusion, and ROIs for the peritumoral region were drawn on the slide showing most T2 hyperintensity as correlated with the T2-weighted series and Fluid Attenuation Inversion Recovery (FLAIR) series. If perfusion data did not help to delineate the aforementioned tumor regions, T2, FLAIR and/or T1 post-contrast images were used as these are used in daily clinical practice to detect regions suspected for either early tumor progression or pseudoprogression. For each scan, a total of 6 ROIs were independently drawn by the three investigators who were all blinded to histopathological outcomes and clinical data at time of assessment. Consequently, a total of 18 ROIs per DSC-MR scan were obtained (Fig. 2).

### Statistical analysis

The statistical analyses were performed by using IBM SPSS Statistics version 26 for Windows, (*SPSS Inc., Chicago, IL, USA*). The intraclass correlation coefficient (ICC) was used to calculate the inter-observer variability between the perfusion metrics as assessed by three researchers. The ICC is scored according to the classification as proposed by Cicchetti; ICC 0.00–0.20 poor, 0.21–0.40 fair, 0.41–0.60 moderate, 0.61–0.80 good and 0.81–1.00 excellent [24]. A two-way random ICC model, with absolute agreement, single measures and a 95% confidence interval was used. The perfusion parameters of the differently shaped ROIs in each specific region (i.e., whole tumor, tumor hotspot and peritumoral region) were averaged for further statistical assessment. The perfusion metrics as assessed by the circular and the polygonal ROI were compared by use of the dependent T-test.

## Results

Table 1 provides information with regard to the demographics of the included patients. Table 2 provides an overview of the mean perfusion metrics per group. Below, we provide more specific information with regard to perfusion metrics and the impact of the differently shaped ROIs in the different subregions.

**Table 1:** Characteristics of included patients and gliomas

Group	Total (*n* = 40)	Pre-treatment group (*n* = 20)	Post-treatment group (*n* = 20)
Mean age at imaging ± standard deviation (years)	61 ± 13.6	61 ± 13.2	60 ± 13.4
Gender (n, %)			
F	*n* = 24, 60%	*n* = 13, 65%	*n* = 11, 55%
M	*n* = 16,40%	*n* = 7, 35%	*n* = 9, 45%
Histologically confirmed diagnosis (n, %)	WHO II: 8, 20% WHO III: 2, 5% WHO IV: 30, 75%	WHO II: 4, 20% WHO III: 1, 5% WHO IV: 15, 75%	WHO II: 4, 20% WHO III: 1, 5% WHO IV: 15, 75%

**Table 2:** Mean CBF, rCBV and MTT values by three observers in two groups of patients

	**CBF** (mL/100 g/min)	**CBV** (mL/100 g)	**MTT** (sec)
**Pre-treatment group**			
*Mean*	70,45	4,03	3,54
*Median*	66,83	3,11	2,90
*Std. Deviation*	26,16	2,16	2,05
*Range*	97,74	14,35	13,28
*Minimum*	22,26	1,51	1,36
*Maximum*	120,00	15,85	14,64
**Post-treatment group**			
*Mean*	52,39	2,92	3,51
*Median*	47,92	2,17	3,19
*Std. Deviation*	27,38	1,84	1,59
*Range*	110,54	7,46	10,08
*Minimum*	9,46	0,39	1,39
*Maximum*	120,00	7,85	11,47

*CBV* Cerebral blood volume, *ICC* Intraclass correlation coefficient, *CBF* Cerebral blood flow, *TTP* Time-to-peak

### Mean values of the circular ROIs

Mean values (± SD) of CBF, CBV, and TTP values derived from the circular ROIs covering the whole tumor region in untreated patients were 99.8 mL/100 g/min (± 30.1); 6.7 mL/100 g (± 1.3); and 4.4 s (± 5.7), respectively. Mean values (± SD) of CBF, CBV, and TTP values derived from the circular ROIs covering the peritumoral edema in untreated patients were 55.2 mL/100 g/min (± 23.4); 2.8 mL/100 g (± 1.1); and 2.9 s (± 1.8), respectively. Mean values (± SD) of CBF, CBV, and TTP values derived from the circular ROIs covering the tumor hotspot in untreated patients were 58.7 mL/100 g/min (± 37.3); 2.6 mL/100 g (± 1.5); and 3.3 s (± 2.0), respectively.

Mean values (± SD) of CBF, CBV, and TTP values derived from the circular ROIs covering the whole tumor in post-treatment patients were 83.1 mL/100 g/min (± 35.7); 5.4 mL/100 g (± 2.3); and 3.8 s (± 3.1), respectively. Mean values (± SD) of CBF, CBV, and TTP values derived from the circular ROIs covering the peritumoral edema in post-treatment patients were 38.4 mL/100 g/min (± 26.4); 1.9 mL/100 g (± 1.1); and 3.1 s (± 1.9), respectively. Mean values (± SD) of CBF, CBV, and TTP values derived from the circular ROIs covering the tumor hotspot in post-treatment patients were 35.7 mL/100 g/min (± 30.1); 1.6 mL/100 g (± 1.0); and 3.5 s (± 1.8), respectively.

### Mean values of the polygonal ROIs

Mean values (± SD) of CBF, CBV, and TTP values derived from the polygonal ROIs covering the whole tumor region in untreated patients were 94.5 mL/100 g/min (± 27.4); 6.5 mL/100 g (± 3.6); and 4.1 s (± 3.4), respectively. Mean values (± SD) of CBF, CBV, and TTP values derived from the polygonal ROIs covering the peritumoral edema in untreated patients were 57.0 mL/100 g/min (± 25.1); 2.9 mL/100 g (± 1.1); and 3.1 s (± 1.9), respectively. Mean values (± SD) of CBF, CBV, and TTP values derived from the polygonal ROIs covering the tumor hotspot in untreated patients were 57.6 mL/100 g/min (± 32.4); 2.5 mL/100 g (± 1.2); and 3.4 s (± 2.4), respectively.

Mean values (± SD) of CBF, CBV, and TTP values derived from the polygonal ROIs covering the whole tumor region in post-treatment patients were 79.3 mL/100 g/min (± 34.7); 5.0 mL/100 g (± 1.8); and 4.3 s (± 3.9), respectively. Mean values (± SD) of CBF, CBV, and TTP values derived from the polygonal ROIs covering the peritumoral edema in post-treatment patients were 42.5 mL/100 g/min (± 30.2); 2.1 mL/100 g (± 1.3); and 2.9 s (± 1.5), respectively. Mean values (± SD) of CBF, CBV, and TTP values derived from the polygonal ROIs covering the tumor hotspot in post-treatment patients were 35.4 mL/100 g/min (± 25.6); 1.6 mL/100 g (± 0.9); and 3.5 s (± 1.8), respectively.

### Comparing circular- and polygonal ROIs in different regions

In untreated patients, when comparing the impact of ROI shape on mean perfusion metrics, it was found that mean CBF, CBV and TTP values of the whole tumor regions were not significantly different between groups (*P* = 0.082; *P* = 0.597 and *P* = 0.367, respectively). Mean CBF, CBV and TTP values were not found statistically different in untreated patients when using different ROI shapes in the peritumoral edema (*P* = 0.341; *P* = 0.465 and *P* = 0.755, respectively). When comparing mean perfusion metrics of the tumor hotspot as assessed by either circular ROIs or polygonal ROIs, no statistical significant difference was seen for CBF, CBV and TTP in the untreated patients (*P* = 0.509; *P* = 0.957 and *P* = 0.498, respectively).

In post-treatment patients, ROI shape did not impact mean CBF, CBV and TTP values in the whole tumor region (*P* = 0.08; *P* = 0.375 and *P* = 0.189, respectively). In the peritumoral edema of post-treatment patients, the use of circular and polygonal ROIs did not result in different CBF, CBV and TTP values (*P* = 0.243; *P* = 0.328 and *P* = 0.285, respectively). No statistical significant differences were observed between different ROIs in the tumor hotspot region with regard to CBF CBV or TTP inpost-treatment patients (*P* = 0.460; *P* = 0.557 and *P* = 0.825).

### Intraclass correlation coefficient of ROIs of the pre-treatment patient group

Circular ROIs covering the whole tumor region, the tumor hotspot and the peritumoral region showed to have a significant overlap between observers with regard to CBV values (*P* < 0.0001; *P* = 0.003 and *P* < 0.001, respectively). When assessing the CBF values with circular ROIs, a significant overlap was found in the whole tumor region (*P* < 0.0001) and the peritumoral region (*P* < 0.001). The circular ROIs placed at the tumor hotspot, however, provided non-significant overlap when used to assess CBF (*P* = 0.058). When using circular ROIs to assess the TTP at the tumor hotspot, a non-significant overlap was observed (*P* = 0.269). In the whole tumor region and peritumoral region, the circular ROIs provided significant overlapping TTP values (*P* < 0.0001 and *P* < 0.001, respectively).

Polygonal ROIs covering the whole tumor region revealed significant overlap between observers for CBF, CBV and TTP values (*P* = 0.029; *P* = 0.001; *P* < 0.001, respectively). The polygonal ROIs of the tumor hotspot showed that for CBF and CBV, the ICC-values were significantly overlapping (*P* < 0.001; *P* = 0.021, respectively), whereas the TTP-values were non-similar (*P* = 0.268). Polygonal ROIs covering the peritumoral region showed to have non-similar CBF, CBV and TTP values between readers (*P* = 0.057; *P* = 0.078; *P* = 0.363, respectively).

Detailed information with regard to the ICC-values is provided in Table 3.

**Table 3:** Inter-observer classification for perfusion parameters of differently shaped regions of interest and in different areas in glioma patients prior to treatment

	*Perfusion metric*	*Intraclass Correlation*	*95%-Confidence interval*		*P-value*
			Lower Bound	Upper Bound	
Whole tumor region; Untreated group of patients; Circular ROI	CBF	0.963	0.924	0.984	** < 0.001**
	CBV	0.948	0.892	0.977	** < 0.001**
	TTP	0.951	0.899	0.979	** < 0.001**
Tumor hotspot; Untreated group of patients; Circular ROI	CBF	0.441	-0.156	0.756	0.058
	CBV	0.501	-0.032	0.782	**0.030**
	TTP	0.198	-0.658	0.649	0.269
Peritumoral region; Untreated group of patients; Circular ROI	CBF	0.821	0.630	0.922	** < 0.001**
	CBV	0.924	0.843	0.967	** < 0.001**
	TTP	0.741	0.465	0.887	** < 0.001**
Whole tumor region; Untreated group of patients;Polygonal ROI	CBF	0.504	-0.026	0.783	**0.029**
	CBV	0.687	0.352	0.863	**0.001**
	TTP	0.856	0.703	0.937	** < 0.001**
Tumor hotspot; Untreated group of patients; Polygonal ROI	CBF	0.828	0.644	0.925	** < 0.001**
	CBV	0.529	0.027	0.794	**0.021**
	TTP	0.200	-0.655	0.650	0.268
Peritumoral region; Untreated group of patients; Polygonal ROI	CBF	0.442	-0.154	0.756	0.057
	CBV	0.409	-0.223	0.742	0.078
	TTP	0.113	-0.834	0.612	0.363

*CBV* Cerebral blood volume, *ICC* Intraclass correlation coefficient, *CBF* Cerebral blood flow, *TTP* Time-to-peak

### Intraclass correlation coefficient of ROIs of the post-treatment patient group

Circular ROIs covering the whole tumor region showed to have significant overlap between CBF and CBV values between the readers (*P* = 0.017; *P* < 0.001, respectively). The TTP-values, on the other hand, were non-similar between readers when using circular ROIs covering the whole tumor region (*P* = 0.143). The circular ROIs of the tumor hotspot showed that CBF, CBV and TTP values were different between readers (*P* = 0.700; *P* = 0.776; *P* = 0.671, respectively). In addition, circular ROIs covering the peri-tumoral region also resulted in non-similar CBF, CBV and TTP values (*P* = 0.497; *P* = 0.529; *P* = 0.505, respectively).

Polygonal ROIs covering the whole tumor region provided non-similar CBF, CBV and TTP values (*P* = 0.503; *P* = 0.397; *P* = 0.448, respectively). The polygonal ROIs of the tumor hotspot showed no significant overlap between CBF, CBV and TTP values (*P* = 0.506; *P* = 0.565; *P* = 0.377,respectively). Polygonal ROIs covering the peri-tumoral region also showed non-similar CBF, CBV and TTP values (*P* = 0.513; *P* = 0.514; *P* = 0.564, respectively).

Detailed information with regard to the ICC-values is provided in Table 4.

**Table 4:** Inter-observer classification for perfusion parameters of differently shaped regions of interest and in different areas in glioma patients in the post-treatment group

	*Perfusion metric*	*Intraclass Correlation*	*95%-Confidence interval*		*P-value*
			Lower Bound	Upper Bound	
Whole tumor region; Post-treatment group of patients; Circular ROI	CBF	0.555	0.062	0.810	**0.017**
	CBV	0.720	0.410	0.880	** < 0.001**
	TTP	0.331	-0.409	0.715	0.143
Tumor hotspot; Post-treatment group of patients; Circular ROI	CBF	-0.260	-1.653	0.462	0.700
	CBV	-0.389	-1.926	0.407	0.776
	TTP	-0.218	-1.566	0.480	0.671
Peritumoral region; Post-treatment group of patients; Circular ROI	CBF	-0.014	-1.137	0.567	0.497
	CBV	-0.048	-1.207	0.553	0.529
	TTP	-0.023	-1.155	0.563	0.505
Whole tumor region; Post-treatment group of patients; Polygonal ROI	CBF	-0.021	-1.150	0.564	0.503
	CBV	0.083	-0.932	0.609	0.397
	TTP	0.034	-1.034	0.588	0.448
Tumor hotspot; Post-treatment group of patients; Polygonal ROI	CBF	-0.025	-1.158	0.563	0.506
	CBV	-0.087	-1.290	0.563	0.565
	TTP	0.102	-0.891	0.617	0.377
Peritumoral region; Post-treatment group of patients; Polygonal ROI	CBF	-0.086	-1.288	0.536	0.564
	CBV	-0.032	-1.174	0.559	0.514
	TTP	-0.032	-1.173	0.560	0.513

*CBV* Cerebral blood volume, *ICC* Intraclass correlation coefficient, *CBF* Cerebral blood flow, *TTP* Time-to-peak

### Comparing the intraclass correlation coefficient of ROIs placed by the inexperienced readers vs. the more experienced reader in the pretreatment setting

Circular ROIs covering the perilesional edema, whole tumor region and tumor hotspot showed to have high ICC-values for measuring CBF values when comparing inexperienced and experienced readers (ICC = 0.852, ICC = 0.592 and ICC = 0.870 respectively). In this set-up, when measuring CBV metrics, we found ICC-values of 0.849, 0.535, and 0.867 in the peritumoral edema, whole tumor region and tumor hotspot, respectively. With regard to TTP measurements with circular ROIs by inexperienced and experienced readers, an ICC of 0.876, 0.601 and 0.885 in peritumoral edema, whole tumor region and tumor hotspot, respectively.

With regard to polygonal ROIs, CBF measurements in the perilesional edema, whole tumor region and tumor hotspot yielded ICCs of 0.885, 0.808, and 0.839, respectively, when comparing inexperienced and experienced readers. In the same setting, polygonal ROIs to measure CBV values in the perilesional edema, whole tumor region and tumor hotspot yielded ICCs of 0.876, 0.780, and 0.882, respectively. TTP values in this set-up were found to yield ICCs of 0.808, 0.557, and 0.785 in the perilesional edema, whole tumor region and tumor hotspot, respectively.

### Comparing the intraclass correlation coefficient of ROIs placed by the inexperienced readers vs. the more experienced reader in the post-treatment setting

Circular ROIs covering the perilesional edema and tumor hotspot showed to have high ICC-values for measuring CBF values between inexperienced and experienced readers (ICC = 0.752 and ICC = 0.529 respectively). Measuring CBF values in the whole tumor region provided fair ICC results (ICC = 0.310) when comparing inexperienced and experienced readers in this study. Circular ROIs covering the peritumoral edema and whole tumor region showed an ICC of 0.488 and 0.511 when comparing inexperienced and experienced readers measuring CBV. When placed in the tumor hotspot, circular ROIs measuring CBV yielded an ICC of 0.236. With regard to TTP measurements with circular ROIs by inexperienced and experienced readers, an ICC of 0.375, 0.762 and 0.043 in peritumoral edema, whole tumor region and tumor hotspot, respectively.

With regard to polygonal ROIs, CBF measurements in the perilesional edema, whole tumor region and tumor hotspot yielded ICCs of 0.654, 0.288, and 0.339, respectively, when comparing inexperienced and experienced readers. In the same setting, polygonal ROIs to measure CBV values in the perilesional edema, whole tumor region and tumor hotspot yielded ICCs of 0.542, 0.210, and 0.024, respectively. TTP values in this set-up were found to yield ICCs of 0.532, 0.133, and 0.043 in the perilesional edema, whole tumor region and tumor hotspot, respectively.

## Discussion

This study showed that readers with varying experience can deliver largely consistent DSC perfusion metrics in untreated glioma patient when working with a well-established image-review protocol. Consistency and DSC perfusion metrics were not impacted by shape of the placed ROI. Based on our findings, the most robust method to assess DSC-MR images in untreated patients comprised the use of circular ROIs and the usage of the CBV metric. With regard to the stability of tumor subregions, the whole tumor region and peritumoral region were considered to be consistently identified, whereas the results derived from the tumor hotspot varied more.

ROI-placement in the DSC-MR perfusion images of post-treatment patients, on the other hand, delivered highly variable results. In this group, shape of the ROIs did not further deteriorate ICC-values. In this group, the ICC-values of the circular ROI covering the whole tumor region provided the most optimal results with significant overlap between the three readers.

It was found, however, that specific tumor subregions yielded more consistent results as compared to other regions. These findings partially contradict the available literature, as it has been stated that polygonal ROIs improve accuracy of determining perfusion metrics [25]. Other groups also found that polygonal ROIs yielded the greatest inter-observer agreement as compared to circular ROIs [26–28].

Regarding the deterioration of ICC values in the post-treatment group, this could be explained by the post-operative changes with regard to neuroanatomy, complicating interpretation of DSC MR perfusion images. A similar trend was observed by Barboriak et al. in dynamic contrast enhanced (DCE) MR perfusion imaging. They found that ICC-values for specific DCE metrics ranged from 0.90 to 1.00 when assessing baseline gliomas. However, the ICC-values of the posttreatment examinations deteriorated significantly and ranged from 0.48 to 0.76 [29]. This induces a dilemma in daily radiological practices as, following the European Association for Neuro-Oncology (EANO) guidelines, the role of DSC-MR perfusion imaging is to monitor efficacy of neurosurgical resection, radiotherapy and/or pharmacotherapy, or as surveillance imaging method after completion of the treatment [30]. Different readers could therefore interpret the same DSC-MR perfusion imaging scan differently, undermining the diagnostic effectiveness of, for example, distinguishing progression from pseudo-progression. This is especially important as developing more objective methods to obtain reproducible data is increasingly important in neuro-oncology imaging, especially when performing multicenter clinical trials. This could be of pivotal importance when using DSC perfusion imaging as a marker of angiogenesis to determine the effects of antiangiogenic treatment [31].

In the reading room, on the other hand, the present findings could also have a relevant impact. Research on MR perfusion in neuro-oncology focuses on the use of brain tumor vascularization and perfusion to predict WHO subtype and molecular diagnostics. In most studies, ROIs are drawn by different readers, independently [32–34]. Due to the high ICC-values in the non-treated gliomas, one could argue that independent placement of ROIs by different observers, which is considered common practice in perfusion weighted imaging studies, is unnecessary. Another method to increase ICC-values in glioma perfusion research, could include the (semi-)automatic segmentation of the ROI. A recent publication on this topic showed that use of semiautomatic segmentation of contrast-enhancing tumor led to increased ICC and a lower coefficient of variation between readers [35].

The current study is partially limited by the inclusion of gliomas with different WHO grades. However, the majority of the included cases concerned glioblastoma (WHO grade IV), which limits the inclusion heterogeneity. Another limitation of the current study concerns its relative small sample size for each group and retrospective nature. One of the strengths of this paper concerns the inclusion of a matched posttreatment group which enables comparison of ICC-values of the pretreatment and posttreatment groups. These insights underline that measuring perfusion metrics in the clinical, often post-operative setting can be difficult and remain operator-dependent. A well-validated assessment protocol should therefore be established. Another strength concerns the use of differently shaped ROIs covering various areas of the tumor region, aiding in taking the first steps of establishing a well-validated assessment protocol for DSC-MR perfusion imaging in the clinical setting. Based on these findings, the authors would recommend different readers to assess post-operative DSC-MR perfusion imaging. However, pre-operative images could be assessed by one reader only.

## Conclusion

Even inexperienced readers can provide consistent DSC perfusion metrics in pre-treatment glioma patients when using a well-established image-review protocol. However, in post-treatment glioma patients, double reading is warranted due to the high inconsistency of assessed DSC perfusion metrics. This study further showed that the ROI shape of preference can be chosen without impacting the quantified DSC perfusion metric assessment.

## Data Availability

Completely anonymized data can be acquired upon reasonable request by contacting the corresponding author. Data which could comprise patient anonymity will not be made available for others.

## Abbreviations

CBF: Cerebral blood flow
CBV: Cerebral blood volume
DSC-MRI: Dynamic susceptibility contrast magnetic resonance imaging
ROI: Region of interest
FLAIR: Fluid attenuated inversion recovery
ICC: Intra-class coefficient
MTT: Mean transit time
TTP: Time to peak
